# Oral Dysphagia and Its Associations With Nutrition, Physical Functions, and Depression for the Elderly in the Community

**DOI:** 10.1093/geroni/igaa057.777

**Published:** 2020-12-16

**Authors:** Yinan Zhao, Hongting Ning, Lulu Liao, Hengyu Hu, Huijing Chen, Hui Feng

**Affiliations:** 1 central south university, Changsha, China; 2 Central South University, Changsha, China

## Abstract

Objectives: To explore the occurrence of oral dysphagia problems among elderly in community and to investigate whether the severity of oral dysphagia problems is associated with nutrition, physical functions and mental health. Methods: Trained nurses conducted a rigorous assessment of 679 elderly people living in communities in Zhuzhou. Basic personal information, The Short Form Mini Nutritional Assessment (MNASF), Activities of daily living (ADL), Patient Health Questionnaire-9 (PHQ-9) were used to assess each elderly. Oral dysphagia was assessed by means of a standardized questionnaire including clinically symptoms of oral dysphagia such as coughing and choking. Results: A total of 654 old people was enrolled in this study, including 250 (38.2%) developing the one or more symptoms of oral dysphagia, the most common symptoms were: drinking water choking (30.7%), followed by saliva (9.6%), dysphagia or pain (6.4%),food loss from the corner of the mouth (2.3%), foreign body sensation of the esophagus (1.8%),food residue (1.2%).The severity of oral dysphagia problems was linearly associated with nutrition, physical function, depression, education level and current care status. The higher the burden of oral symptoms, the lower the self-rated health. Conclusions: The occurrence of oral dysphagia symptom was associated in a stepwise fashion with nutrition, physical function, depression, education level and current care status. In the community, the early recognition, diagnosis and treatment of oral dysphagia is an important link of reducing the mortality and improving the rehabilitation outcome. Additionally, the psychological construction in the elderly should be taken seriously by their families and community workers.

